# TNF superfamily members promote hepatitis C virus entry via an NF-κB and myosin light chain kinase dependent pathway

**DOI:** 10.1099/jgv.0.000689

**Published:** 2017-04-01

**Authors:** N. F Fletcher, A. R Clark, P Balfe, J. A McKeating

**Affiliations:** ^1^​ Centre for Human Virology, Institute for Immunology and Immunotherapy, University of Birmingham, Birmingham, UK; ^2^​ Institute of Inflammation and Ageing, University of Birmingham, Birmingham, UK; ^†^​ Present address: Nuffield Department of Medicine, University of Oxford, UK.

**Keywords:** hepatitis C, polarized entry, TNF, MLCK

## Abstract

Preventing virally induced liver disease begins with an understanding of the host factors that define susceptibility to infection. Hepatitis C virus (HCV) is a global health issue, with an estimated 170 million infected individuals at risk of developing liver disease including fibrosis and hepatocellular carcinoma. The liver is the major reservoir supporting HCV replication and this hepatocellular tropism is defined by HCV engagement of cellular entry receptors. Hepatocytes are polarized *in vivo* and this barrier function limits HCV entry. We previously reported that activated macrophages promote HCV entry into polarized hepatocytes via a TNF-α-dependent process; however, the underlying mechanism was not defined. In this study, we show that several TNF superfamily members, including TNF-α, TNF-β, TWEAK and LIGHT, promote HCV entry via NF-κB-mediated activation of myosin light chain kinase (MLCK) and disruption of tight junctions. These observations support a model where HCV hijacks an inflammatory immune response to stimulate infection and uncovers a role for NF-κB-MLCK signalling in maintaining hepatocellular tight junctions.

## Introduction

Hepatitis C virus (HCV) is a global health issue with an estimated 170 million infected individuals worldwide. The virus infects hepatocytes in the liver and leads to progressive liver injury, fibrosis and in some cases hepatocellular carcinoma. Infection is initiated by the viral encoded E1E2 glycoproteins interacting with cellular receptors: tetraspanin CD81, scavenger receptor BI (SR-BI), epidermal growth factor receptor, the cholesterol absorption regulator Niemann–Pick disease type C1-like 1 protein, and tight junction proteins claudin-1 and occludin (reviewed in [1, 2]). This multi-step process of receptor engagement primes a clathrin-dependent endocytic uptake of particles into the liver.

Hepatocytes are polarized and HCV particles entering the liver encounter their basolateral surface that expresses the ‘viral attachment’ proteins CD81 and SR-BI [3]. We previously reported the presence of CD81-claudin-1 complexes at the basolateral hepatocellular membrane and live cell imaging demonstrated HCV-dependent CD81 and claudin-1 co-endocytosis and fusion with Rab5 expressing endosomes, supporting a role for this receptor complex in HCV internalization [4]. In contrast, occludin localizes almost exclusively to the apical tight junctions, which are considered inaccessible to virus particles, raising questions on its role in the viral internalization process [5, 6].

Many viruses infect polarized epithelia during host invasion and, given the well-recognized function of the epithelium to restrict macromolecule movement across mucosa, viruses have evolved diverse strategies to overcome this barrier [7, 8]. HCV is associated with liver inflammation during chronic infection, and we previously reported that IL-1β and TNF-α secreted from activated macrophages promote HCV entry by disrupting hepatocyte tight junctions; however, the mechanism by which this occurred was undefined [9]. Here, we demonstrate that the TNF superfamily members TNF-α, TNF-β, TWEAK and LIGHT promote HCV infection through an NF-κB- and myosin light chain kinase (MLCK)-dependent pathway. These data highlight a role for HCV exploiting TNF superfamily signalling responses in the liver to promote viral entry and persistence.

## Results

### TNF superfamily members promote HCV infection of polarized hepatocytes

**Fig. 1.**
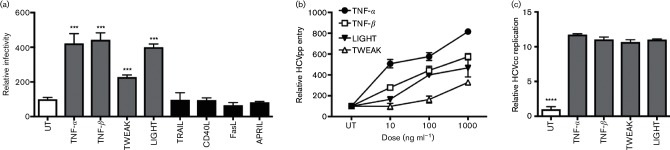
TNF superfamily members promote HCV infection of polarized HepG2 cells. (a) HepG2.CD81 cells were polarized for 5 days and infected with HCVpp in the presence or absence of TNF superfamily members TNF-α, TNF-β, TWEAK, LIGHT, TRAIL, CD40L, FasL or APRIL at 100 ng ml^−1^ and incubated for 72 h. Cytokines that promote HCV entry are depicted by light grey bars, cytokines that had no detectable effect are depicted by black bars and the untreated control (UT) is depicted by a white bar. (b) Polarized HepG2.CD81 cells were treated with an increasing dose of TNF-α, TNF-β, TWEAK or LIGHT and infected with HCVpp for 72 h. (c) TNF-α, TNF-β, TWEAK and LIGHT (100 ng ml^−1^) increased cell culture propagated hepatitis C virus (HCVcc) J6/JFH infection of polarized HepG2.CD81 cells, where infection was assessed by qRT (quantitative reverse transcriptase)-PCR quantitation of viral RNA levels after 48 h. Data are presented as mean infectivity ±sd relative to a UT and are representative of three independent experiments where: ****, *P*<0.0001; ***, *P*<0.001.

We previously reported that TNF-α promotes HCV entry into polarized hepatocytes [9]. TNF-α is secreted by a wide range of activated immune cells and earlier studies showed that the pro-viral activity of conditioned media from lipopolysaccharide-activate macrophages was only partially inhibited by neutralizing TNF-α, suggesting additional pro-viral factors [9]. To investigate the potential for other TNF superfamily members to promote hepatocellular permissiveness to support HCV entry, we screened a panel of recombinant human TNF superfamily members for their effect on HCV pseudoparticle (HCVpp) infection of polarized HepG2.CD81 cells (Fig. 1a). In addition to TNF-α, we found that TNF-β, TWEAK and LIGHT promote HCVpp infection in a dose-dependent manner (Fig. 1b). In contrast, TNF family members TRAIL, CD40L, FasL and APRIL had no detectable effect on HCVpp infection despite reports of HepG2 cells expressing their cognate receptors [10–13]. We confirmed that TNF-α, TNF-β, TWEAK and LIGHT increased native HCV J6/JFH infection of polarized HepG2 cells and observed a significant increase in viral RNA 48 h post-infection (Fig. 1c).

**Fig. 2.**
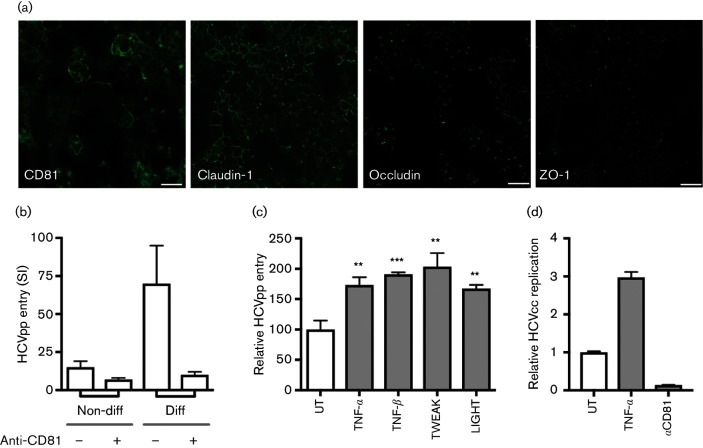
TNF promotes HCV infection of differentiated HepaRG cells. (a) HCV receptor staining of differentiated HepaRG cells show basolateral CD81 and claudin-1 expression. The tight junction proteins occludin and ZO-1 were restricted to apical BC-like structures of hepatocyte-like cells. Bars, 10 µm. (b) Non-differentiated and differentiated HepaRG cells were infected with HCVpp in the presence or absence of anti-CD81 2.131 (10 µg ml^−1^); data is presented as Specific Infectivity (SI) reflecting the mean HCVpp luciferase values relative to an envelope deficient control pseudoparticle. (c) TNF-α, TNF-β, TWEAK and LIGHT (100 ng ml^−1^) promote HCVpp infection of differentiated HepaRG cells. (d) TNF-α (100 ng ml^−1^) increased HCVcc J6/JFH infection of differentiated HepaRG cells, where infection was assessed by qRT-PCR determination of viral RNA levels after 72 h. As a control, anti-CD81 2.131 (10 µg ml^−1^) neutralized HCV infection of differentiated cells is shown. Data are presented as mean infectivity ±sd relative to the untreated control (UT) and are representative of two independent experiments where: ***, *P*<0.001; **, *P*<0.01.

The hepatocyte-derived HepG2 cell line has provided a valuable *in vitro* model for studying hepatocyte polarity [14]. The bi-potent hepatocyte progenitor HepaRG clone can be differentiated into mixed cultures of polarized hepatocytes and cholangiocytes [15], and provide an independent model to validate our results. Differentiated HepaRG-derived hepatocyte-like cells expressed the essential HCV entry factors CD81, occludin and claudin-1, together with the junctional protein ZO-1, which localize to bile canalicular (BC)-like structures (Fig. 2a). Consistent with this pattern of viral receptor expression, differentiated HepaRG cells supported HCVpp entry that was neutralized with an anti-CD81 antibody, whereas HCVpp failed to infect the non-differentiated progenitor cells (Fig. 2b). Using HCVpp expressing a GFP reporter, we could demonstrate that HCVpp entry was restricted to hepatocyte-like cells, whereas Vesicular Stomatitis Virus encoded G expressing pseudoparticle (VSV-Gpp) infected cells with both hepatocyte and cholangiocyte morphology (data not shown). These observations were consistent with our previous report that primary cholangiocytes were not permissive for HCV infection [16]. Importantly, TNF-α, TNF-β, TWEAK and LIGHT increased the permissiveness of differentiated HepaRG cells to support HCVpp infection (Fig. 2c). Furthermore, pre-treating differentiated HepaRG cells with TNF (100 ng ml^−1^) induced a significant increase in native HCV RNA levels that was CD81-dependent (Fig. 2d). Collectively, these data demonstrated that several TNF superfamily members promote HCV entry into polarized hepatocytes.

### TNF superfamily members disrupt tight junction integrity and promote HCV infection through activation of NF-κB p65/RelA

**Fig. 3.**
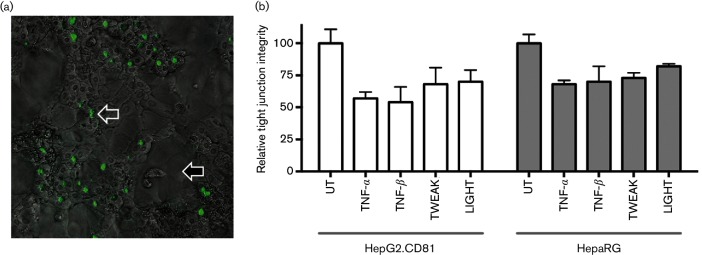
TNF superfamily members disrupt tight junctions. (a) Differentiated HepaRG cultures comprised hepatocytes (open arrow) and cholangiocytes (filled arrow). Magnification, ×400. Cultures were treated with the fluorescent dye CMFDA that binds the apical transporter multidrug resistance-associated protein 2 enabling real time imaging of bile canaliculi (green structures). Treating cultures with CMFDA (30 min) identified BC structures. (b) Tight junction integrity of HepG2.CD81 and HepaRG cells was measured by quantifying the number of BC structures retaining CMFDA following 1 h cytokine treatment (100 ng ml^−1^) in five independent fields of view at ×10 magnification with data expressed relative to untreated (UT) cells. *N*=3 independent experiments.

To investigate the mechanism by which TNF superfamily members promote HCV entry, we first assessed their effect on BC tight junction integrity. The fluorescent dye 5-chloromethylfluorescein diacetate (CMFDA), which binds the apical transporter multidrug resistance-associated protein 2 (MRP2), enabled real-time imaging of BC structures in differentiated HepaRG (Fig. 3a) and HepG2 cells, and allowed us to measure the effect of cytokines on tight junction integrity [5]. TNF-α, TNF-β, TWEAK and LIGHT disrupted tight junction integrity in both model systems (Fig. 3b), with no detectable effect on cellular viability. Of note, all cytokine-mediated effects on HepG2 polarity were reversible and tight junction integrity was restored over a 24 h period after their removal, demonstrating the plasticity of the trafficking pathways regulating tight junction proteins.

**Fig. 4.**
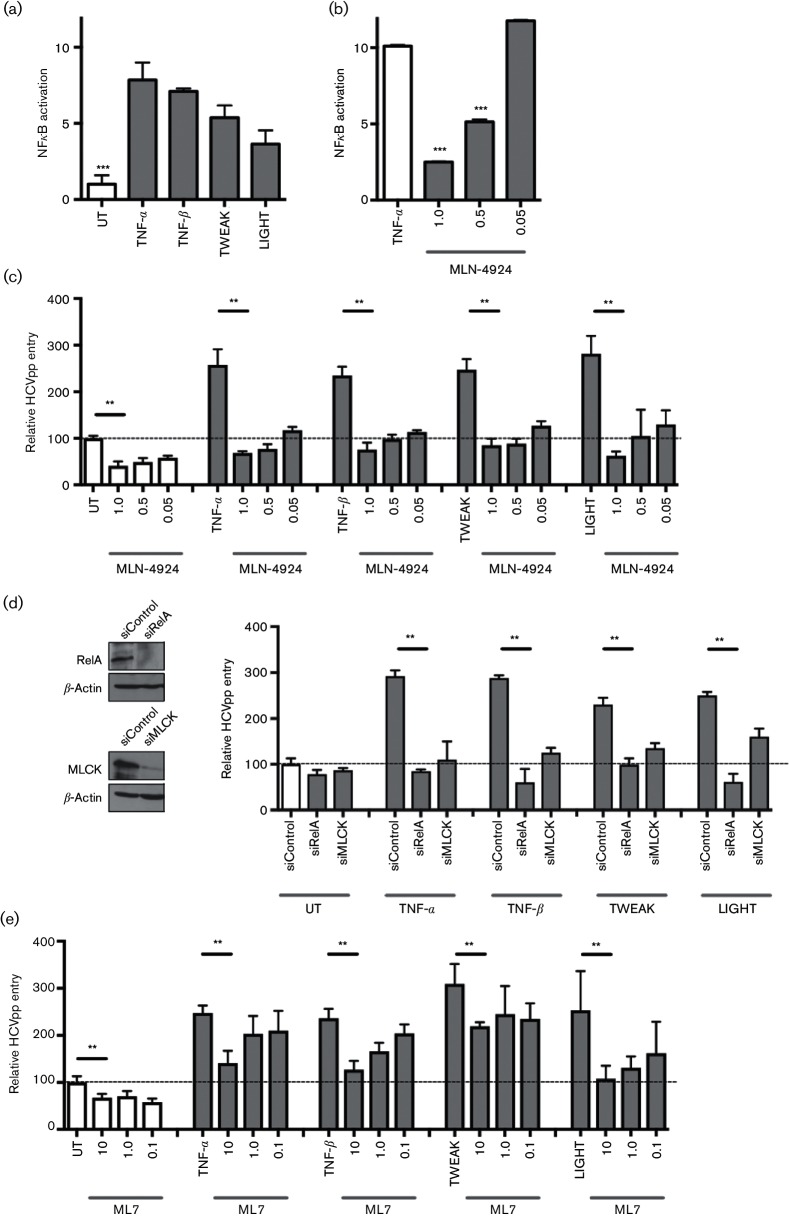
TNF superfamily members promote HCV entry in an NF-κB- and MLCK-dependent manner. (a) HepG2.CD81 cells expressing an NF-κB luciferase reporter construct were allowed to polarize for 5 days prior to treating with TNF family members (100 ng ml^−1^) for 24 h. Luciferase reporter activity is expressed relative to UT cells. (b) Polarized HepG2.CD81 cells expressing NF-κB reporter were treated with an increasing dose of MLN-4924 (µg ml^−1^) in the presence of TNF-α (100 ng ml^−1^). (c) Polarized HepG2.CD81 cells were treated with the indicated concentrations of MLN-4924 and 100 ng cytokine ml^−1^, 15 min prior to infecting with HCVpp; data is presented as mean infectivity ±sd relative to the UT cells (represented by a dotted line). (d) HepG2.CD81 cells were transfected with control siRNA (siControl), siRelA or siMLCK for 48 h prior to treating with 100 ng ml^−1^ of the indicated cytokines and infecting with HCVpp. Western blots demonstrate knockdown of RelA or MLCK, respectively. (e) Polarized HepG2.CD81 cells were treated with the MLCK inhibitor, ML7, at the indicated concentrations, together with 100 ng cytokine ml^−1^ for 15 min prior to infecting with HCVpp. Data are presented relative to UT cells. Data are representative of three independent experiments, where: ***, *P*<0.001; and **, *P*<0.01. UT, Untreated control.

The TNF superfamily are well recognized to activate NF-κB in a range of cell types and using an NF-κB luciferase reporter assay we showed that TNF-α, TNF-β, TWEAK and LIGHT activate NF-κB in HepG2 cells (Fig. 4a). MLN-4924 inhibits the Nedd8-activating enzyme that regulates cullin-RING ubiquitin ligases [17], and has been reported to limit canonical and non-canonical NF-κB signalling pathways [18, 19]. We used MLN-4924 and RelA shortinterfering RNA (siRNA) to investigate the role of NF-κB in TNF-α, TNF-β, TWEAK and LIGHT promoted HCV infection of polarized hepatocytes. We confirmed that MLN-4924 inhibits TNF-α-induced NF-κB reporter activity in a dose-dependent manner (Fig. 4b). Furthermore, MLN-4924 abrogated the pro-viral activity of TNF superfamily members to promote HCVpp entry (Fig. 4c). It is noteworthy that MLN-4924 inhibited basal non-cytokine-mediated HCVpp infection (Fig. 4c), whereas it had no effect on the permissiveness of non-polarized HepG2 or Huh-7 cells to support HCVpp entry, suggesting a role for NF-κB signalling in polarized HCV entry. Silencing RelA abrogated the pro-viral activity of TNF superfamily members for HCVpp infection (Fig. 4d), demonstrating a role for RelA in TNF-boosted HCV entry.

### TNF superfamily members promote HCV entry in a MLCK-dependent manner

TNF-α disruption of gastrointestinal and lung epithelial permeability is dependent on NF-κB activation of MLCK, which regulates occludin and ZO-1 endocytosis [20–22]. We therefore investigated the role of MLCK in basal and TNF-mediated HCVpp infection. Silencing MLCK reduced the pro-viral activity of TNF superfamily members for HCVpp infection of HepG2.CD81 cells (Fig. 4d).

**Fig. 5.**
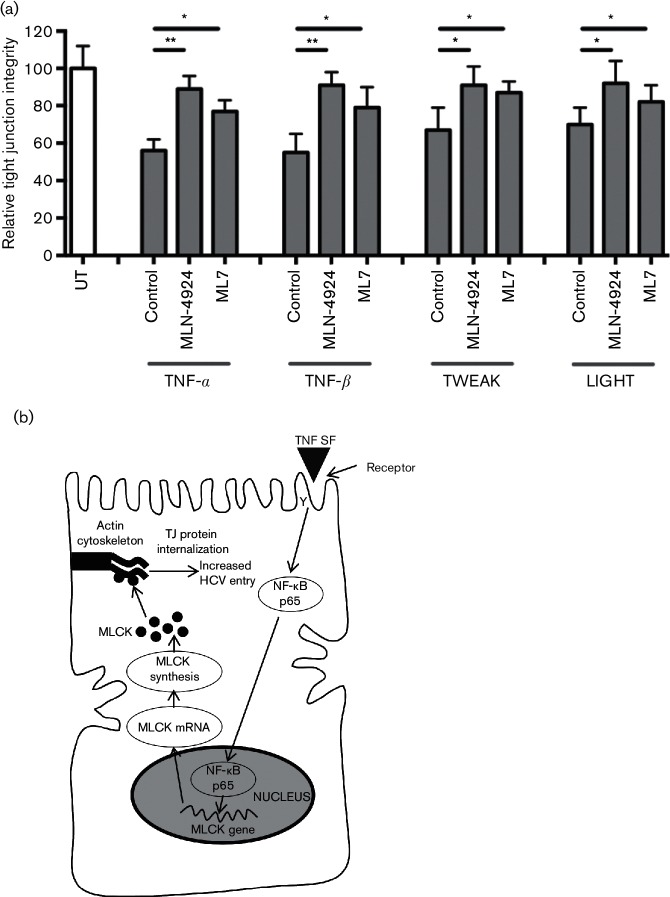
NF-κB- and MLCK-dependent TNF perturbation of tight junction integrity. (a) Polarized HepG2.CD81 cells were incubated with NF-κB RelA inhibitor MLN-4924 (1 µg ml^−1^) or MLCK inhibitor ML7 (10 µg ml^−1^) for 15 min prior to treating with TNF-α, TNF-β, TWEAK and LIGHT (100 ng ml^−1^) for 1 h, and quantifying the number of BC structures retaining CMFDA. Data is presented from a single experiment with triplicate wells and is representative of two independent experiments. UT, Untreated control cells. (b) Proposed model for TNF superfamily members promote HCV uptake into polarized cells. The binding of TNF superfamily members (TNF SF) to their receptors activates NF-κB p65 (RelA), which promotes MLCK transcription and synthesis. We hypothesize that MLCK activates perijunctional actin and trafficking of tight junction (TJ) proteins occludin and ZO-1 to intracellular compartments [20], which facilitate HCV fusion.

Similar results were obtained by treating cells with the MLCK inhibitor, ML7, demonstrating a role for MLCK in TNF-α-, TNF-β-, TWEAK- and LIGHT-dependent HCVpp infection of polarized to HepG2 (Fig. 4e). We noted that ML7 induced a significant reduction of basal HCVpp infection. Finally, we demonstrated that both ML7 and MLN-4924 treatments abrogated the effects of TNF superfamily members on HepG2 tight junction integrity (Fig. 5a). These data confirm that TNF superfamily members perturb hepatocellular tight junctions via RelA- and MLCK-dependent pathways that most likely play an important role in maintaining hepatocyte polarity.

## Discussion

Our study shows a role for various members of the TNF superfamily to promote HCV infection by activating NF-kB and MLCK-signalling pathways to reduce hepatocellular tight junction integrity. We observed similar results with two polarizing hepatocyte models, the well-established HepG2 hepatoma cell line and the bi-potent HepaRG progenitor that can differentiate into hepatocytes and cholangiocytes [23]. We [9] and others [24, 25] have reported that HCV can activate macrophages to express TNF-α, enabling the virus to hijack this pro-inflammatory responses to promote its own entry. TNF superfamily members are secreted by activated immune cells, including lymphocytes, macrophages and Kupffer cells, and are highly expressed in the inflamed liver [9, 26], providing an environment that potentiates HCV infection of naive hepatocytes. We previously reported that TNF induced a re-organization of occludin and ZO-1 from apical BC structures in HepG2 cells via an undefined mechanism [9]. Using inhibitors and siRNA silencing approaches, we demonstrate a role for NF-κB and MLCK-signalling in TNF-α, TNF-β, TWEAK and LIGHT enhancement of HCV entry via their perturbation of tight junction integrity. The observation that inhibiting NF-κB and MLCK limits HCVpp infection in the absence of inflammatory mediators highlights a role for these signalling pathways in polarized virus entry (Fig. 5b).

Claudin-1 and occludin play an essential role in defining HCV entry and it is of interest to note that these proteins were initially discovered to regulate HCV internalization into non-polarized human embryonal kidney cells [25, 27]. To date, the published literature support a model where claudin-1 and occludin regulation of HCV internalization is independent of their location at tight junctions [2], raising the question of how TNF superfamily members potentiate HCV infection. We reported that HCV interacts with claudin-1-CD81 complexes at the basolateral hepatocellular membrane [3, 4]. At the present time, there is limited evidence to show that HCV trafficks to the tight junction, indeed Blaising and colleagues demonstrated HCV internalization via the basolateral membrane and localization within Rab5-positive intracellular vesicles [28].

Receptor mobility and lateral diffusion speed in the plasma membrane can regulate the movement of viruses at the plasma membrane and can impact on particle internalization rate(s) [29, 30]. We previously reported that hepatocyte polarization limits CD81 mobility via cytoplasmic tail interactions with the cytoskeleton via Ezin-Radixin-Moesin proteins [31]. Furthermore, HepG2 cells expressing a C-terminal truncated CD81 mutant supported comparable levels of HCV entry independent of polarization status, suggesting that membrane protein dynamics on polarized cells may limit pathogen attachment and infection. Indeed, our report showing that TNF-α increased CD81 lateral mobility in polarized HepG2 cells supports this model [9].

Polarized lung and gut epithelia are well recognized to restrict the invasion of microbial pathogens, and many viruses have evolved strategies to modulate the cortical actin network to promote infection of polarized cells [8]. Adenovirus infection of lung epithelia is potentiated by macrophage expressed IL-8, which stimulates the apical expression of an alternate viral receptor CAR^Ex8^ to enable the infection of intact epithelium [32]. Our previous report that TNF-α promoted the permissivity of polarized HepG2 cells to support entry of pseudoparticles expressing Vesicular stomatitis virus (VSV), measles or Lassa virus glycoproteins is consistent with a role for cytoskeletal changes associated with cell polarization to alter viral receptor trafficking [9].

Like all epithelial cells, hepatocytes mediate the flow of macromolecules via their polarized surface that comprises tight junctions and cell–cell adhesions. However, our understanding of the molecular pathways and inflammatory mediators regulating hepatocyte polarity in health or disease are not well understood [33]. The tight junction is a dynamic structure, and increasing evidence from studies of gut and lung epithelia highlight the role of MLCK in regulating the tight junction [21]. Our study supports a common pathway for TNF family members to regulate hepatocyte polarity via MLCK, as reported for other epithelial cells. In conclusion, we have demonstrated a role for TNF superfamily signalling in regulating HCV entry and persistence.

## Methods

### Cell lines and antibodies

HepG2 cells stably expressing CD81 [5] and 293T were propagated in Dulbecco's Modified Eagle's medium (DMEM) supplemented with 10 % FBS and 1 % non-essential amino acids. HepaRG cells were obtained from Invitrogen and maintained in Williams E medium supplemented with 10 % FBS, 5 µg insulin ml^−1^ and 50 µM hydrocortisone. HepaRG cell differentiation was performed over a 4 week period, and the media supplemented with 2 % DMSO for the final 2 weeks of differentiation, as described elsewhere [23]. All cells were maintained at 37 °C and 5 % CO_2_.

The following primary antibodies were used: anti-CD81 (2.131), anti-claudin-1 mAb (Abnova), anti-claudin-1 polyclonal sera and anti-occludin (Invitrogen). Secondary labelled antibodies, Alexa-488 goat anti-mouse IgG, Alexa-488 goat anti-rabbit IgG, Alexa-594 goat anti-mouse IgG and Alexa-594 goat anti-rat IgG were purchased from Invitrogen. Recombinant human cytokines were obtained from Peprotech, MLN-4924 from Calbiochem and ML7 from Sigma-Aldrich.

### Laser scanning confocal microscopy

HepaRG cells were differentiated and fixed with ice-cold methanol (claudin-1, occludin) or 3 % paraformaldehyde (CD81) 24 h after seeding. Primary antibodies were applied for 1 h at room temperature. After washing twice with PBS, anti-mouse, anti-rabbit or anti-rat Alexa Fluor 488 (Invitrogen) secondary antibody was applied for 1 h at room temperature. Cells were counterstained with DAPI (Invitrogen) for nuclei visualization and mounted with ProLong Gold antifade (Invitrogen). Cells were viewed by laser-scanning confocal microscopy on a Zeiss META head confocal microscope with a 100× oil-immersion objective.

### Quantifying tight junction barrier function

To determine the functionality of tight junctions and whether they restricted paracellular diffusion of solutes from the BC lumen to the basolateral medium, HepG2 or HepaRG cells were incubated with 5 mM CMFDA (Invitrogen) at 37 °C for 10 min to allow translocation to the BC lumen. After washing extensively with PBS, the capacity of BC to retain CMFDA was enumerated using a fluorescence microscope.

### NF-κB reporter assays

HepG2.CD81 cells were transfected with a NF-κB reporter plasmid expressing a firefly luciferase gene (pNiFty-luc; Invivogen) and polarized for 5 days. Cells were treated with TNF superfamily members (100 ng ml^−1^) for 24 h, lysed and luciferase activity measured for 10 s in a luminometer (Lumat LB 9507).

### HCVpp genesis and infection

Pseudoviruses were generated by transfecting 293T cells with plasmids encoding a human immunodeficiency virus provirus expressing luciferase and HCV strain H77 E1E2 region or a no envelope control (Env−), as previously reported [34]. Virus-containing media was added to target cells for 8 h, unbound virus removed and media replaced with DMEM/3 % FBS. At 72 h post-infection, the cells were lysed, luciferase substrate added and luciferase activity measured for 10 s in a luminometer (Lumat LB 9507). Specific infectivity was calculated by expressing mean HCVpp luciferase values relative to the mean Env-pp RLU signal. Cytokines were added to cells at the same time as pseudovirus, and inhibitors were pre-incubated with cells for 15 min prior to the addition of cytokine and pseudovirus.

### Cell culture propagated hepatitis C virus genesis and infection

HCV SA13/JFH was generated and infection quantified by real-time PCR detection of viral RNA, as previously described [9].

### RelA and MLCK silencing and Western blotting

HepG2.CD81 cells were transfected with a pool of siRNAs (Smartpool) directed against RelA or MLCK, or a non-specific control siRNA (Dharmacon). Forty-eight hours post-transfection, cells were treated with TNF superfamily members (100 ng ml^−1^) and infected with HCVpp. In parallel, cells were lysed and RelA and MLCK expression measured as previously described [35] by Western blot using RelA and MLCK antibodies (Cell Signaling Technology).

### Statistical analysis

Results are expressed as the mean ±1 sd of the mean. Statistical analyses were performed using Students *t*-test in Prism 4.0 (GraphPad) with a *P*<0.05 being considered statistically significant and where required corrected for multiple sampling (Bonferroni).
